# Age Affects Quantity but Not Quality of Antibody Responses after Vaccination with an Inactivated Flavivirus Vaccine against Tick-Borne Encephalitis

**DOI:** 10.1371/journal.pone.0034145

**Published:** 2012-03-26

**Authors:** Karin Stiasny, Judith H. Aberle, Michael Keller, Beatrix Grubeck-Loebenstein, Franz X. Heinz

**Affiliations:** 1 Department of Virology, Medical University of Vienna, Vienna, Austria; 2 Institute for Biomedical Aging Research, Austrian Academy of Sciences, Innsbruck, Austria; Centers for Disease Control and Prevention, United States of America

## Abstract

The impairment of immune functions in the elderly (immunosenescence) results in post-vaccination antibody titers that are significantly lower than in young individuals. It is, however, a controversial question whether also the quality of antibodies declines with age. In this study, we have therefore investigated the age-dependence of functional characteristics of antibody responses induced by vaccination with an inactivated flavivirus vaccine against tick-borne encephalitis (TBE). For this purpose, we quantified TBE virus-specific IgG and neutralizing antibody titers in post-vaccination sera from groups of young and elderly healthy adults and determined antibody avidities and NT/ELISA titer ratios (functional activity). In contrast to the quantitative impairment of antibody production in the elderly, we found no age-related differences in the avidity and functional activity of antibodies induced by vaccination, which also appeared to be independent of the age at primary immunization. There was no correlation between antibody avidity and NT/ELISA ratios suggesting that additional factors affect the quality of polyclonal responses, independent of age. Our work indicates that healthy elderly people are able to produce antibodies in response to vaccination with similar avidity and functional activity as young individuals, albeit at lower titers.

## Introduction

The effects of aging on the immune system – commonly termed ‘immunosenescence’ – are multifaceted and impair both innate and adaptive responses [1], [2], [3], [4]. Together, these defects lead to suboptimal immune reactions, associated with an increased susceptibility to infectious diseases and a lower vaccine-effectiveness in the elderly [5], [6], [7]. In many instances, quantitative measurements of serum antibodies – ideally by the use of functional assays such as virus neutralization or bacterial opsonophagocytosis – provide excellent correlates of protection induced by vaccination [8] and there is overwhelming evidence from a large body of immunization studies – both in animals and in humans – that the amount of antibodies induced is strongly reduced and that titers decline more rapidly in older compared to younger individuals [5], [9]. Factors such as deficiencies in T-cell help, diminished germinal center reactions, intrinsic B cell defects, reduced support for long-lived plasma cells in the bone marrow and reduced numbers of total and specific memory B cells all contribute to the quantitative impairment of Ig production in the elderly [9], [10], [11], [12], [13]. However, data on the effects of aging on antibody avidity and the underlying processes of somatic hypermutation, affinity maturation and the selection of high-affinity clones are conflicting [7], [10], [14]. Since these processes can have a profound impact on the functionality of the antibodies induced in the course of active immunization, we have investigated the effect of age on the quality of antibody responses after vaccination with an inactivated tick-borne encephalitis (TBE) vaccine. For this purpose, we performed a quantitative analysis of avidities and functional activities of TBE virus (TBEV)-specific antibodies in post-vaccination sera from cohorts of different age groups.

TBEV is closely related to yellow fever, dengue, West Nile and Japanese encephalitis virus [15] and represents a significant public health problem in large parts of Europe and Asia [16]. Like with other encephalitogenic flaviviruses, a functional humoral immune response is critically important in controlling infections [17] and passively transferred antibodies are fully protective against TBE in an animal model [18]. In humans, the disease can be effectively prevented by vaccination with a formalin-inactivated purified whole-virus vaccine that is in widespread use in countries of Europe and Russia [16], [19]. The immunization schedule consists of a primary vaccination (two doses at an interval of about one month), a third vaccination after one year and further booster vaccinations recommended at varying intervals in different countries [16]. Consistent with the general decline of immune functions [12], previous studies had revealed a significantly lower antibody response in people more than 60 years of age compared to that of younger individuals (<30 years) [20], [21]. Subsequent studies, however, have shown that the antibody response in people between 50 to 60 years old was already as impaired as in people >60 years of age [20].

Despite a strong quantitative impairment of antibody responses in healthy elderly adults our study shows that there is no significant age-dependent difference in the avidities and in the ratios of neutralizing to ELISA-binding activities (functional activities) of antibodies induced by TBE vaccination. These factors were also not affected by the age at primary immunization. Surprisingly, antibody avidity did not correlate with functional activity in both young and old. This ratio, however, was highly variable from person to person and - at least to a certain extent - appears to be an imprinted individual trait, regardless of age.

## Materials and Methods

### Human sera

Two panels of serum samples obtained after vaccination with an inactivated whole-virus vaccine (FSME-Immun® 0.5 ml, Baxter) were used in our study: Panel A was derived from a booster immunization study in young and old healthy adults conducted at the Institute of Biomedical Aging Research, Austrian Academy of Sciences, in Innsbruck and consisted of 79 pre- and post-booster vaccination sera used previously for determining antibody titers [20] as well as 29 additional sera of the same cohort. In total, we therefore had 108 sera in the age groups of <30 (n = 21) and >50 (n = 87; 50–60 years: n = 22; 60–70 years: n = 30; >69 years: n = 35) for functional analyses. All vaccinees had a history of a completed basic immunization and varying numbers of booster vaccinations. Serum was collected prior to booster vaccination and 28 days later. Panel B was derived from a primary immunization study in young and old healthy adults conducted at the Department of Virology, Medical University of Vienna and consisted of sera from 33 individuals in the age groups of <30 (n = 11) and >60 (n = 21). Serum was collected 28 days after the basic immunization and after the first booster immunization.

In both studies, participants had no clinically significant diseases, acute infections or health conditions known to affect immune responses and were not under immunosuppressive therapy [13], [20].

### Ethics statement

The studies were approved by the ethics committees of the Medical University of Innsbruck and of the Medical University of Vienna, respectively, and the participants gave their written informed consent.

### IgG ELISA

TBEV-specific IgG antibodies were analyzed by ELISA using purified formalin-inactivated TBEV as described previously [22]. Sera were analyzed in duplicates and quantified in VIE Units using a standard human anti-TBEV serum arbitrarily set at 1000 VIE Units. ELISA values above 155 VIE Units were considered positive.

### Neutralization Assay

Neutralization tests (NTs) were carried out in baby hamster kidney cells (ATCC BHK-21) as described previously [22]. Serial dilutions of polyclonal sera (in duplicates) were mixed with 25 pfu virus and incubated for 1 h at 37°C. Cells were added and incubation was continued for 3 days. The presence of virus in the supernatant was assessed by ELISA [23]. Titers were determined after curve-fitting using a four-parameter logistic regression and a cut-off of 50% reduction of the ELISA absorbance in the absence of antibody. NT titers ≥10 were considered positive.

### Avidity ELISA

Avidities of TBEV-specific IgG antibodies were analyzed in an ELISA using purified formalin-inactivated TBEV as described previously [22]. Sera were tested in tenfold serial dilutions, including a wash step with or without 8 M urea in PBS pH 7.4 after serum incubation with antigen. Titers were determined at an absorbance value of 1.0 (490 nm) after curve-fitting using a four-parameter logistic regression (GraphPad Prism 5; GrapPad Software Inc.). The avidity of each serum was calculated with the following formula: Avidity (%) = (titer at absorbance 1.0 plus urea/titer at absorbance 1.0 without urea)×100. Three independent experiments were performed for each serum to calculate mean avidities. A post infection serum with low avidity was used as a control in each assay.

### Statistical Analyses

Statistical analyses were performed with GraphPad Prism 5 (GraphPad Software Inc.). Logarithmic transformation of the data was carried out to obtain approximate normal distribution of antibody concentrations, avidity values and NT/ELISA ratios. A sample size of 17 volunteers in each group provides a power of 80% (alpha 0.05) to detect a 10% difference in avidities as well as a 20% difference in NT/ELISA ratios following the formula of Dallal for two-sided statistical tests [24]. Two-tailed t-tests or one-way ANOVA (Tukey post test) were applied to the transformed data for significance testing and correlation coefficients were determined with the Pearson correlation test. P values<0.05 were regarded as statistically significant.

## Results

### Similar IgG antibody avidities in young and elderly vaccinees

To investigate potential age-dependent differences in the quality and functional activity of antibodies induced by vaccination, we first analyzed a serum panel from a TBE booster vaccination study conducted in young and old adults (panel A, Materials and Methods) with no underlying diseases or impairment by any immunosuppressive treatments. Consistent with published data [20], pre-and post-booster antibody concentrations as well as neutralizing antibody titers were significantly lower among those aged >50 years (range: 51–87 years of age) than in the young control group aged <30 years (Figure S1).

**Figure 1:**
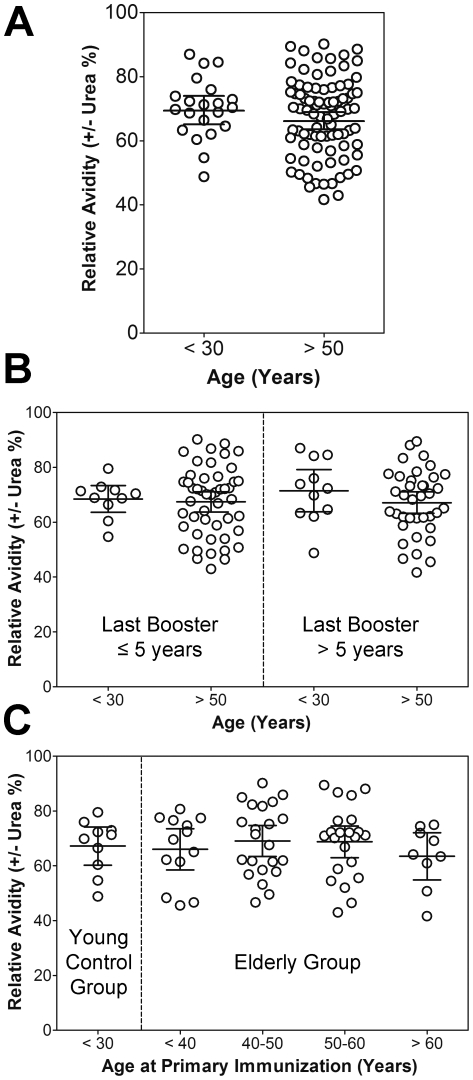
Avidity of TBEV-specific IgG antibodies of young (<30) and elderly (>50: range 51–87 years of age) TBE vaccinees after booster immunization. Avidities were determined by ELISA after a urea-washing step and expressed as percent of the values obtained in the absence of urea. (A) Groups were based on age at booster vaccination. (B) Groups were based on age at booster vaccination (young, elderly) and on the time interval to the last vaccination before booster. (C) Groups were based on age at booster vaccination (young, elderly) and the elderly vaccinees were further stratified according to their age at primary vaccination which was known for 76 of 108 samples. Data represent mean values +/−95% confidence intervals.

Avidity is considered to be a crucial parameter of the quality and functionality of antibodies [17], [25] and reports on its age-dependent impairment are controversial [7], [14]. We therefore determined the relative avidities of post-booster sera using an IgG ELISA combined with a urea wash step (Materials and Methods). As shown in Figure 1A, the avidities ranged from ca. 40 to 90% which are characteristic values for a booster antibody response and higher than those observed in patients with a recent TBEV infection (mean avidity 20%) [22]. With respect to the mean avidities, no statistically significant difference was observed between the two age groups (unpaired t-test; P value = 0.28). In addition, we specifically compared the avidities of the oldest vaccinees (>69 years) to those <30 years of age and also in that case no statistically significant difference was found between the two groups (unpaired t-test; P value = 0.26; data not shown).

To assess a possible influence of i) the time period before booster and ii) the age at primary immunization on IgG avidities, elderly subjects (range: 51 to 87 years of age at booster vaccination) were stratified by the interval since the last TBE vaccination (≤5 and >5years) (Figure 1B) as well as the age at primary immunization (<40, 40–50, 50–60, >60 years) (Figure 1C). As can be seen from the figures, neither the time point of the last vaccination (ANOVA; P value = 0.71) nor the age at primary immunization had an impact on IgG avidities (ANOVA; P value = 82).

### Similar functional activities of antibodies in young and elderly vaccinees

**Figure 2:**
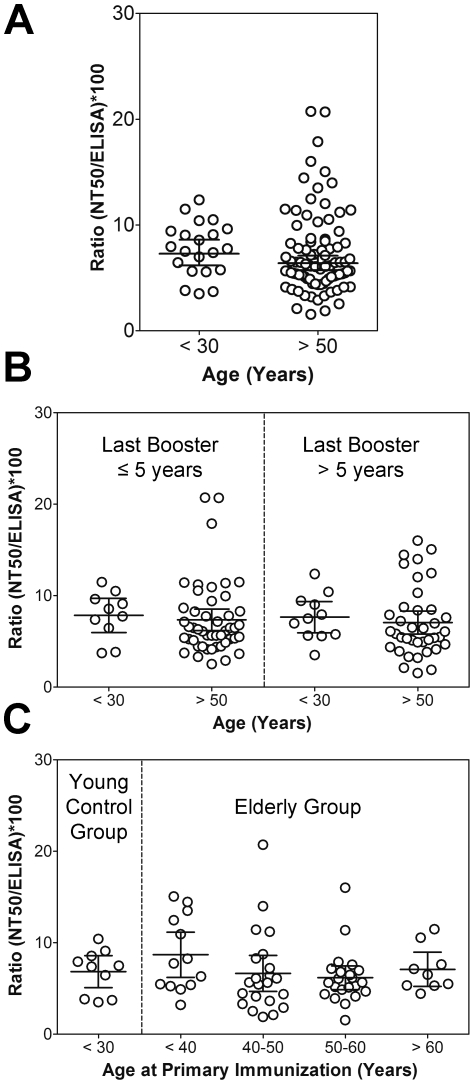
Functional activities of TBEV-specific IgG antibodies of young (<30) and elderly (>50: range 51–87 years of age) TBE vaccinees after booster immunization. The functional activity was measured by calculating the ratio of neutralizing antibodies to ELISA binding IgG antibodies according to the following formula: (NT50 titer/ELISA IgG units)×100. (A) Groups were based on age at booster vaccination. (B) Groups were based on age at booster vaccination (young, elderly) and on the time interval to the last vaccination before booster. (C) Groups were based on age at booster vaccination (young, elderly) and the elderly vaccinees were further stratified according to their age at primary vaccination which was known for 76 of 108 samples. Data represent mean values +/−95% confidence intervals.

The key quality of protective antiviral antibodies is their capacity to bind to epitopes involved in virus neutralization. Infection and vaccination, however, can induce substantial amounts of antibodies that are measurable in in vitro binding assays (such as ELISA) but do not contribute to virus neutralization and protection [26], [27]. To evaluate the functional quality of all of the virus-reactive antibodies induced by vaccination and to compare this parameter between the young and the elderly, we determined the ratios of neutralizing antibody titers (measured in NT) versus total TBEV-specific ELISA binding IgG antibodies. Similar to what has been demonstrated for antibody avidity, no significant differences were detected in the NT/ELISA ratios between the two age groups (Figure 2A) (unpaired t-test; P value = 0.25) and there was no significant difference between the oldest group (>69 years of age) and those of <30 years of age (unpaired t-test; P = 0.42; data not shown). Also, neither the time point of the last vaccination before the booster (Figure 2B) (ANOVA; P value = 0.61) nor the age at primary immunization (Figure 2C) (ANOVA; P value = 0.29) had an influence on the NT/ELISA ratios suggesting that the overall functionality of antibodies is not affected by the age of the vaccinee.

**Figure 3:**
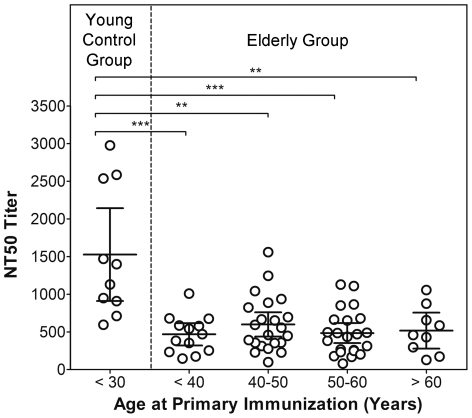
TBEV-specific neutralizing antibody titers of young (<30) and elderly (>50: range 51–87 years of age) TBE vaccinees after booster immunization. Groups were based on age at booster vaccination (young, elderly) and the elderly vaccinees were further stratified according to their age at primary vaccination which was known for 76 of 108 samples. Data represent mean values +/−95% confidence intervals. Statistically significant differences between pairs of groups (P values of <0.05) are indicated by lines and asterisks (ANOVA; Tukey post test).

Although age at primary immunization did not appear to influence the quality of antibody responses, we analyzed whether it had an effect on the amount of booster-induced TBEV neutralizing antibodies. As shown in Figure 3, the antibody titers of the elderly booster groups were similar and no statistical difference was found (ANOVA; Tukey post hoc test; P values>0.05). The titers of the elderly, however, were significantly reduced in comparison to those of the young control group (ANOVA; Tukey post hoc test; P values<0.05).

### No correlation between antibody avidities and functional quality of antibodies

We could not detect differences in the mean IgG avidities and NT/ELISA ratios between the young and elderly, but some degree of variation of both parameters was observed in both age groups (Figures 1 and 2). Since affinity maturation is regarded as essential for the generation of highly functional antibodies [17], we would have expected co-variation of the two parameters. Surprisingly, however, analysis of the data did not reveal any significant correlation (Figure S2; Pearson correlation cofefficients r = 0.06 and r = −0.14 in the young and elderly, respectively), suggesting that other factors in addition to avidity are major determinants of functional antibody activity in this case.

### Tendency of individual imprinting of functional antibody activities

**Figure 4:**
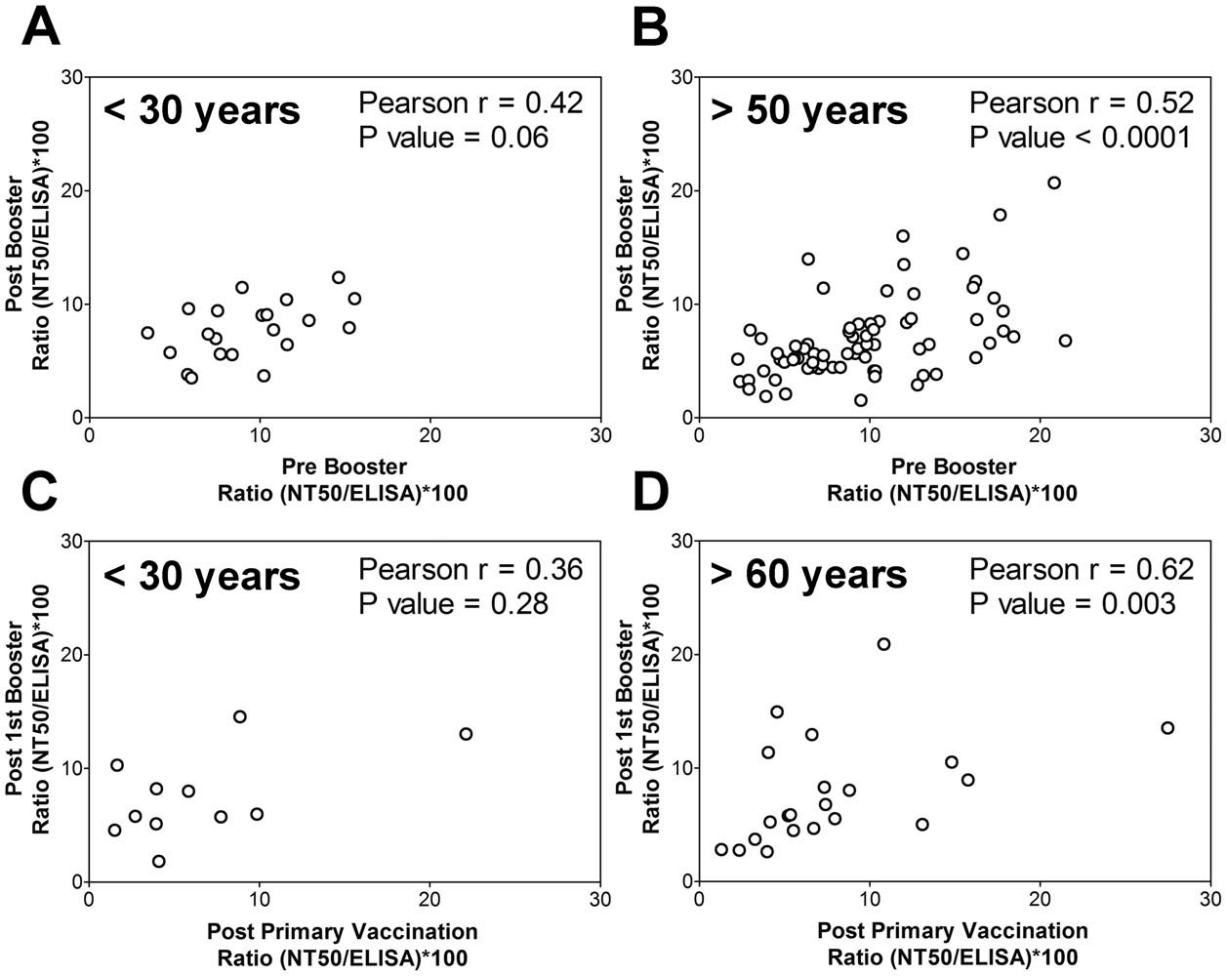
Correlation of NT/ELISA ratios pre- and post-TBE booster vaccination (A,B) as well as after primary and first booster vaccination (C,D) in the young (A,C) and elderly (B,D). The Pearson correlation coefficients r and the P values are indicated.

The strong variation of functional antibody activities (NT/ELISA ratios) observed (Table 1) raised the question whether this trait has the potential to change upon revaccination or has a tendency for individual imprinting. In both age groups, a positive correlation between the pre- and post-booster NT/ELISA ratios was found, indicating that this functional parameter is maintained upon booster vaccination at least to a certain extent (Figure 4A,B).

**Table 1:** Variation of NT/ELISA ratios within groups of young and elderly TBE vaccinees.

Age groups	n	Range of NT/ELISA ratios	Coefficient of variation (%)
<30 years	21	3.5–12.4	32.5
>50 years	87	1.5–20.7	54

To determine whether such a phenomenon can already be detected after primary immunization, we analyzed a second panel of sera from a recent TBE immunization study in which the age-dependence of the immune response after primary vaccination (2 doses) and the first booster vaccination (3rd vaccination) was investigated (panel B, Materials and Methods). Similar to what was seen after booster vaccinations, the comparison of NT/ELISA ratios of sera collected 4 weeks after basic immunization and 4 weeks after the third immunization yielded a positive correlation in the young and elderly groups (Figure 4C,D).

With both serum panels, the correlation in the elderly was slightly stronger and – in contrast to the young – significant. Since, especially in the primary immunization study (Figure 3C), fewer sera of young vaccinees were available for correlation analysis, we combined the data of the young of both studies (Figure 4A,C). Again a positive association was observed and the Pearson coefficient remained in the same range (r = 0.39), but now the correlation was significant (P value = 0.03).

## Discussion

Central to our work was the question whether the quality of antibody responses to an inactivated whole virus vaccine is impaired in healthy elderly. The data presented demonstrate that neither the avidity of the polyclonal antibodies present in TBE post-vaccination sera nor their functional activity (defined as ratio of neutralizing vs. virion binding antibodies) displayed any statistically significant differences in healthy young and old individuals. With respect to avidities, all sera from both age groups had low avidities consistent with values characteristic of robust anamnestic immune responses [22]. The NT/ELISA ratios, however, exhibited strong age-independent individual variation and it might be possible that subtle differences between age groups could go unnoticed due to the relatively small sample size of some groups used in the analyses. The unimpaired quality of antibodies is in clear contrast to the well-established fact – reconfirmed in our study (Figure S1; Figure 3) – that the quantity of post-vaccination antibodies produced in the elderly is severely diminished [5], [6], [28]. Even when primary immunization occurred at a younger age, the antibody titers of elderly vaccinees were significantly reduced compared to those of the young control group.

In the case of TBE vaccination, this impairment was already found for the age group of 50 to 60 year old individuals [20]. In a recent study with the same vaccine, it was further shown that not only the number of antigen-specific memory B cells induced by primary immunization was about 3-fold lower in the elderly but also the antibody response per memory B cell after revaccination was strongly reduced [13]. These impairments were associated with profound functional defects in antigen-specific CD4+helper T cells.

In addition to quantity, however, it is the quality of antibodies that matters and high avidity binding to the pathogen is considered to be essential for antibody effector functions [17], [25]. Data on the influence of age on affinity maturation are conflicting [7], [14], [29], [30] and our analyses of post-vaccination sera suggest that the elderly – despite their overall impairment of immune functions – are still able to produce high-affinity antibodies that have similar functional activities as those produced in young age. These findings are fully congruent with a recent study that analyzed the avidities of plasmablast-derived antibody-responses to seasonal influenza vaccination in young and elderly adults [31]. Although the frequency of vaccine-specific plasmablasts and the amount of antibodies produced by these cells in vitro were lower in the elderly, neither the yields of secreted IgG per plasmablast nor the avidity of these antibodies was different in the two age groups. The annual influenza infections/vaccinations with a variety of subtypes and strains, however, make it difficult to differentiate between primary and booster responses, and exposure history as well as original antigenic sin phenomena can influence the results obtained [31], [32]. Our data, in contrast, are not confounded by such problems of antigenic shift and drift and demonstrate in a non-variable single antigen system that the quality of vaccine-induced antibodies is not diminished in elderly vaccinees and even appears to be unaffected when priming occurs at an advanced age.

It is a surprising and intriguing finding of our work that there was no correlation between antibody avidity and functional activity of vaccine-induced antibodies, a trait that was characteristic for both the young and the old age groups. Considering the importance of high-avidity for antibody effector functions [17], [25], we have to conclude that there are other factors that contribute to their functional activity. These factors may be related to individual differences in the antibody subsets of polyclonal immune responses measured in NT and ELISA. For flaviviruses there is evidence that the most potent virus-neutralizing antibodies are primarily directed to one of the three structural domains (Domain III) of the viral envelope protein E whereas those to other domains are less effective [33]. In the context of NT/ELISA ratios, it is also important to note that the ELISA – due to partial denaturation of the antigen during coating – detects antibodies to antigenic sites that are not exposed at the surface of infectious virions and therefore do not contribute to virus neutralization [26], [27], [34]. Nevertheless, such antibodies can have a profound effect on the ratio of neutralizing to ELISA binding antibodies in individual sera. The importance of these considerations is underscored by studies with monoclonal antibodies obtained after measles and influenza vaccination which revealed that only a relatively small subset of the antibodies reactive in ELISA was able to neutralize the viruses [35]. Therefore, independent of antibody avidity, the composition of antibody populations in individual sera and their varying specificities with subsets of antigenic sites may have a significant impact on their neutralizing activity.

Although our analyses did not reveal an overall difference in the avidities and functional activities (NT/ELISA ratios) of post-vaccination antibodies in young and old, we observed a remarkable degree of individual variation of the ratios in both age groups. We interpret these findings as evidence for variations in the composition of antibody subsets as well as their specificities and functionalities between different individuals. Most interestingly, the functional quality of antibody responses appears to be imprinted individually, at least to a certain extent, since we found a positive correlation between the NT/ELISA ratios before and after booster vaccination and also between primary immunization and first booster vaccination. This suggests a tendency for maintaining a ‘specific antibody profile established during initial priming of the immune response. We have recently shown in a related flavivirus model that certain prime-boost regimens can modulate the ratio of post-vaccination antibody specificities and increase the amount of highly functional antibodies [36]. Specifically designed studies will be necessary to find out to which extent the individual variation of vaccine-induced antibody specificities contributes to the variation in NT/ELISA ratios and possibly to the protectiveness of the antibody response.

## Supporting Information

Figure S1 Mean TBEV-specific IgG units (A) and neutralizing antibody titers (B) of young (<30) and elderly (>50: range 51–87 years of age) TBE vaccinees. Dotted bars: values obtained before TBE booster immunization; white bars: values obtained 4 weeks after TBE booster vaccination. IgG antibodies were determined by ELISA and the functional activity by neutralization tests, using 50% virus neutralization as a cut-off (NT50). Results are expressed as geometric mean values +/−95% confidence intervals. The statistical analyses are given on top of each panel (unpaired t-tests) and P values of <0.05 are significant.
(TIF)

Figure S2 Correlation of avidity and NT/ELISA ratio after TBE booster vaccination in young (A) and elderly (B) individuals. The Pearson correlation coefficients r and the P values are indicated.
(TIF)

## References

[1] Aw D, Silva AB, Palmer DB (2007). Immunosenescence: emerging challenges for an ageing population.. Immunology.

[2] Grubeck-Loebenstein B, Della Bella S, Iorio AM, Michel JP, Pawelec G (2009). Immunosenescence and vaccine failure in the elderly.. Aging Clin Exp Res.

[3] Cancro MP, Hao Y, Scholz JL, Riley RL, Frasca D (2009). B cells and aging: molecules and mechanisms.. Trends Immunol.

[4] McElhaney JE, Effros RB (2009). Immunosenescence: what does it mean to health outcomes in older adults?. Curr Opin Immunol.

[5] Weinberger B, Herndler-Brandstetter D, Schwanninger A, Weiskopf D, Grubeck-Loebenstein B (2008). Biology of immune responses to vaccines in elderly persons.. Clin Infect Dis.

[6] Chen WH, Kozlovsky BF, Effros RB, Grubeck-Loebenstein B, Edelman R (2009). Vaccination in the elderly: an immunological perspective.. Trends Immunol.

[7] Frasca D, Diaz A, Romero M, Landin AM, Blomberg BB (2011). Age effects on B cells and humoral immunity in humans.. Ageing Res Rev.

[8] Siegrist CA, Plotkin SA, Orenstein WA, Offit PA (2008). Vaccine immunology.. Vaccines.

[9] Frasca D, Blomberg BB (2009). Effects of aging on B cell function.. Curr Opin Immunol.

[10] Siegrist CA, Aspinall R (2009). B-cell responses to vaccination at the extremes of age.. Nat Rev Immunol.

[11] Dunn-Walters DK, Ademokun AA (2010). B cell repertoire and ageing.. Curr Opin Immunol.

[12] Weiskopf D, Weinberger B, Grubeck-Loebenstein B (2009). The aging of the immune system.. Transpl Int.

[13] Aberle JH, Stiasny K, Kundi M, Heinz FX (2012). Mechanistic insights into the impairment of memory B cells and antibody production in the elderly.. Age (Dordr).

[14] Blomberg BB, Frasca D (2011). Quantity, not quality, of antibody response decreased in the elderly.. J Clin Invest.

[15] Simmonds P, Becher P, Collett MS, Gould EA, Heinz FX, King AMQ, Lefkowitz E, Adams MJ, Carstens EB (2011). Family Flaviviridae.. Virus Taxonomy IXth Report of the International Committee on Taxonomy of Viruses.

[16] WHO (2011). Vaccines against tick-borne encephalitis: WHO position paper.. Wkly Epidemiol Rec.

[17] Pierson TC, Fremont DH, Kuhn RJ, Diamond MS (2008). Structural insights into the mechanisms of antibody-mediated neutralization of flavivirus infection: implications for vaccine development.. Cell Host Microbe.

[18] Kreil TR, Maier E, Fraiss S, Eibl MM (1998). Neutralizing antibodies protect against lethal flavivirus challenge but allow for the development of active humoral immunity to a nonstructural virus protein.. J Virol.

[19] Heinz FX, Holzmann H, Essl A, Kundi M (2007). Field effectiveness of vaccination against tick-borne encephalitis.. Vaccine.

[20] Weinberger B, Keller M, Fischer KH, Stiasny K, Neuner C (2010). Decreased antibody titers and booster responses in tick-borne encephalitis vaccinees aged 50–90 years.. Vaccine.

[21] Rendi-Wagner P, Paulke-Korinek M, Kundi M, Wiedermann U, Laaber B (2007). Antibody persistence following booster vaccination against tick-borne encephalitis: 3-year post-booster follow-up.. Vaccine.

[22] Stiasny K, Holzmann H, Heinz FX (2009). Characteristics of antibody responses in tick-borne encephalitis vaccination breakthroughs.. Vaccine.

[23] Heinz FX, Tuma W, Guirakhoo F, Kunz C (1986). A model study of the use of monoclonal antibodies in capture enzyme immunoassays for antigen quantification exploiting the epitope map of tick-borne encephalitis virus.. J Biol Stand.

[24] Sexton SA, Ferguson N, Pearce C, Ricketts DM (2008). The misuse of ‘no significant difference’ in British orthopaedic literature.. Ann R Coll Surg Engl.

[25] Klasse PJ, Sattentau QJ (2002). Occupancy and mechanism in antibody-mediated neutralization of animal viruses.. J Gen Virol.

[26] Pulendran B, Ahmed R (2011). Immunological mechanisms of vaccination.. Nat Immunol.

[27] Hangartner L, Zinkernagel RM, Hengartner H (2006). Antiviral antibody responses: the two extremes of a wide spectrum.. Nat Rev Immunol.

[28] Hainz U, Jenewein B, Asch E, Pfeiffer KP, Berger P (2005). Insufficient protection for healthy elderly adults by tetanus and TBE vaccines.. Vaccine.

[29] de Bruijn IA, Remarque EJ, Jol-van der Zijde CM, van Tol MJ, Westendorp RG (1999). Quality and quantity of the humoral immune response in healthy elderly and young subjects after annually repeated influenza vaccination.. J Infect Dis.

[30] Zheng B, Han S, Takahashi Y, Kelsoe G (1997). Immunosenescence and germinal center reaction.. Immunol Rev.

[31] Sasaki S, Sullivan M, Narvaez CF, Holmes TH, Furman D (2011). Limited efficacy of inactivated influenza vaccine in elderly individuals is associated with decreased production of vaccine-specific antibodies.. J Clin Invest.

[32] Kim JH, Skountzou I, Compans R, Jacob J (2009). Original antigenic sin responses to influenza viruses.. J Immunol.

[33] Diamond MS, Pierson TC, Fremont DH (2008). The structural immunology of antibody protection against West Nile virus.. Immunol Rev.

[34] Stiasny K, Kiermayr S, Holzmann H, Heinz FX (2006). Cryptic properties of a cluster of dominant flavivirus cross-reactive antigenic sites.. J Virol.

[35] Pinna D, Corti D, Jarrossay D, Sallusto F, Lanzavecchia A (2009). Clonal dissection of the human memory B-cell repertoire following infection and vaccination.. Eur J Immunol.

[36] Zlatkovic J, Stiasny K, Heinz FX (2011). Immunodominance and functional activities of antibody responses to inactivated West Nile virus and recombinant subunit vaccines in mice.. J Virol.

