# Supplementary figures and images for: Age Affects Quantity but Not Quality of Antibody Responses after Vaccination with an Inactivated Flavivirus Vaccine against Tick-Borne Encephalitis
Source: PLoS One. 2012 Mar 26;7(3):e34145. doi: 10.1371/journal.pone.0034145 (PMC3312914; doi:10.1371/journal.pone.0034145)

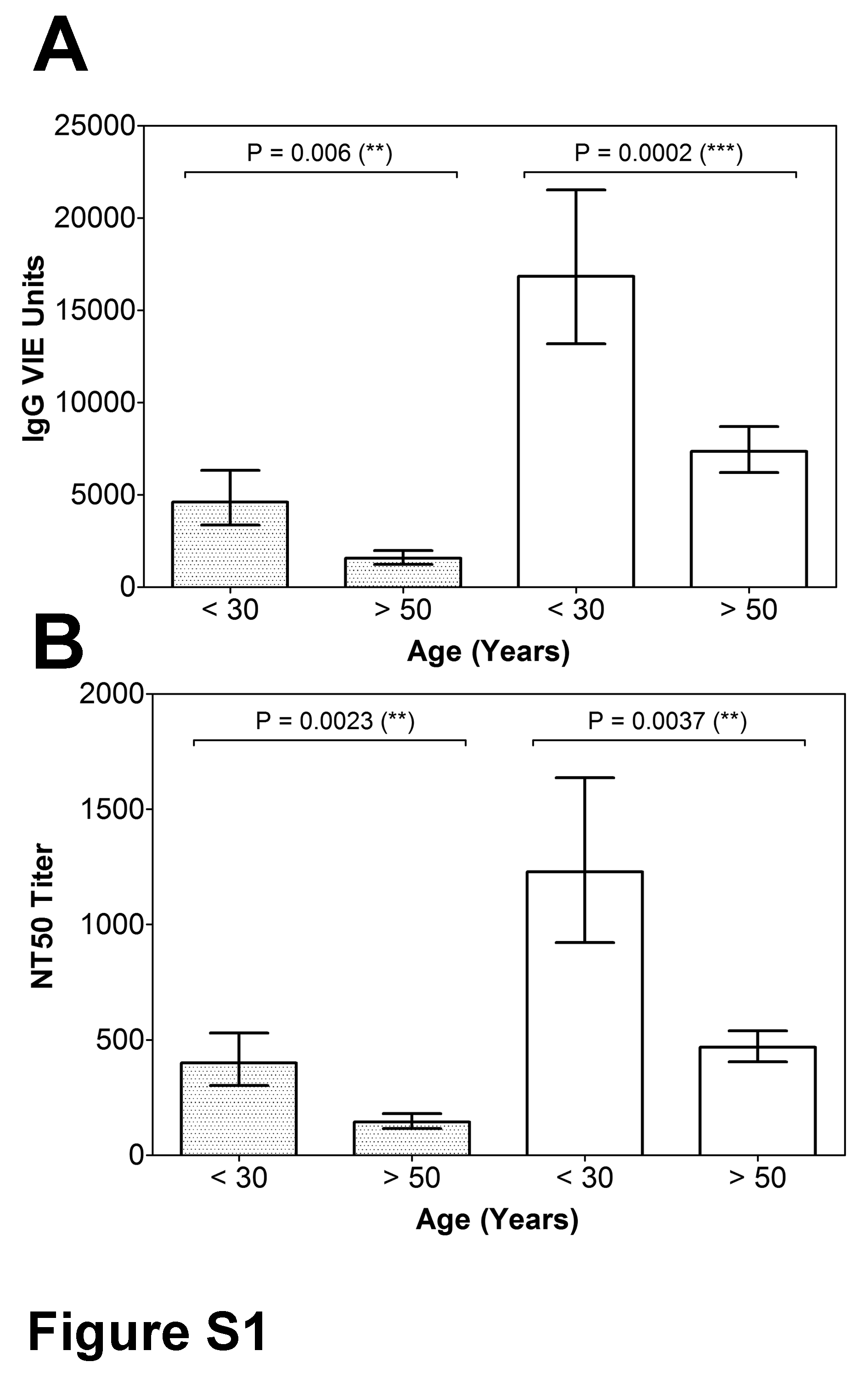

Supplement: Figure S1 — Mean TBEV-specific IgG units (A) and neutralizing antibody titers (B) of young (<30) and elderly (>50: range 51–87 years of age) TBE vaccinees. Dotted bars: values obtained before TBE booster immunization; white bars: values obtained 4 weeks after TBE booster vaccination. IgG antibodies were determined by ELISA and the functional activity by neutralization tests, using 50% virus neutralization as a cut-off (NT50). Results are expressed as geometric mean values +/−95% confidence intervals. The statistical analyses are given on top of each panel (unpaired t-tests) and P values of <0.05 are significant. (TIF) [file pone.0034145.s001.tif]

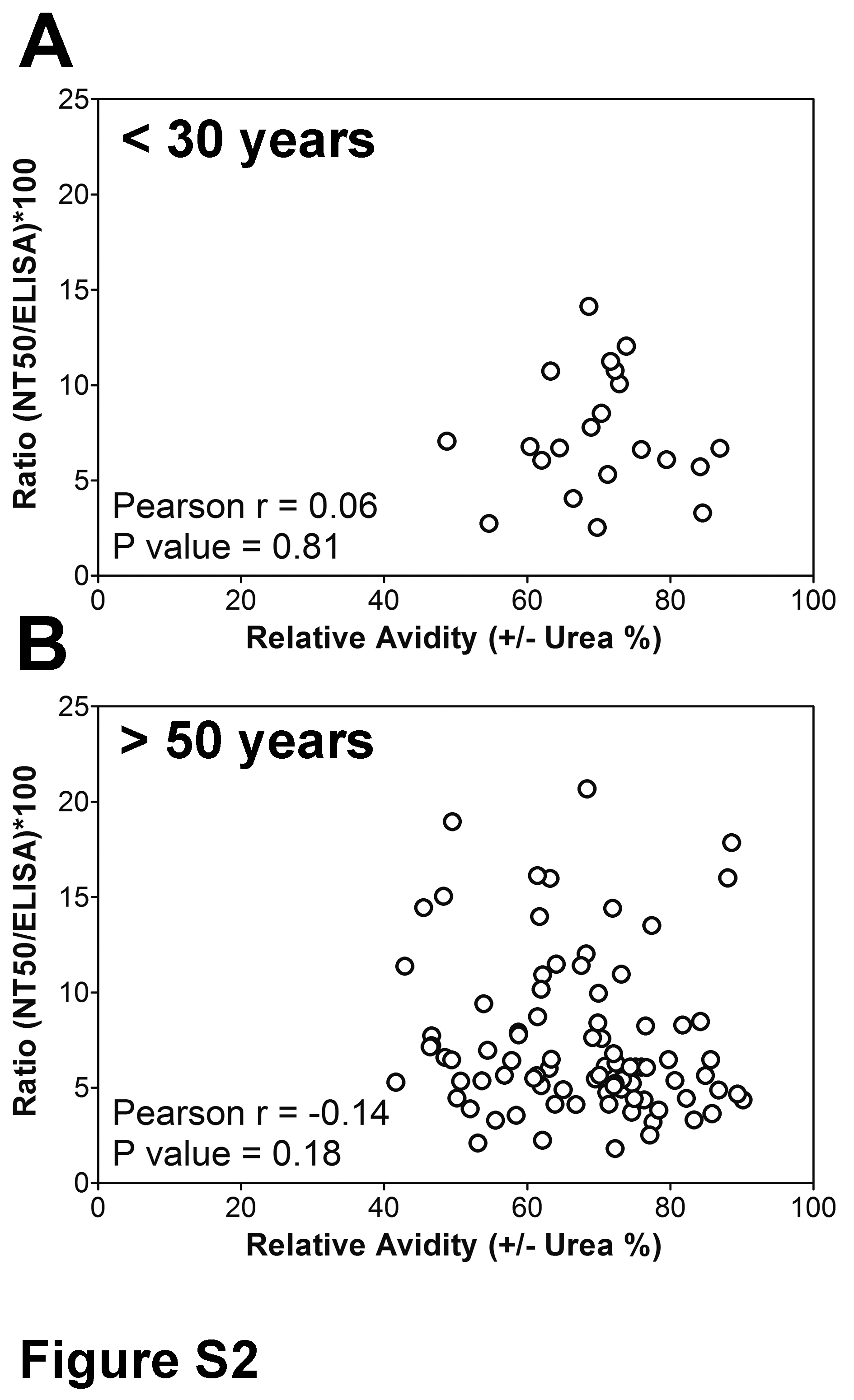

Supplement: Figure S2 — Correlation of avidity and NT/ELISA ratio after TBE booster vaccination in young (A) and elderly (B) individuals. The Pearson correlation coefficients r and the P values are indicated. (TIF) [file pone.0034145.s002.tif]
